# Commentary: Using goal-driven deep learning models to understand sensory cortex

**DOI:** 10.3389/fncom.2018.00004

**Published:** 2018-01-19

**Authors:** Qiulei Dong, Hong Wang, Zhanyi Hu

**Affiliations:** ^1^National Laboratory of Pattern Recognition, Institute of Automation, Chinese Academy of Sciences, Beijing, China; ^2^University of Chinese Academy of Sciences, Beijing, China; ^3^CAS Center for Excellence in Brain Science and Intelligence Technology, Beijing, China

**Keywords:** goal-driven deep learning models, hierarchical convolutional neural network, IT neuron, categorization, convergent features

Recently, a goal-driven modeling approach of sensory cortex is proposed in Yamins and DiCarlo (2016). The basic idea of this approach is to first optimize a hierarchical convolutional neural network (HCNN) for performing an ethologically relevant task, then once the network parameters have been fixed, to compare the outputs of different layers of the network to neural data. The success of this approach is exemplified by the results in Yamins et al. (2014), where a 4-layer HCNN, called HMO, was used to predict IT neuron spikes on image object stimuli. Notably by only optimizing the 8-way image categorization performances, not only can the top output layer of the HMO quantitatively predict IT neuron responses, but its penultimate layer can also automatically predict V4 neuron responses. In Hong et al. (2016), under the same approach, a 6-layer HCNN was trained on ImageNet (Russakovsky et al., 2015) (a benchmark dataset for image object categorization in the computer vision field, containing 1.3 million category-labeled training images of 1,000 different categories) to successfully predict category-orthogonal object properties along the ventral stream. Another demonstrative example is the work in Khaligh-Razavi and Kriegeskorte (2014), showing that when the 10-category representational dissimilarity matrices were used together with the outputs of all the 8 layers of the AlexNet in Krizhevsky et al. (2012), called the IT-geometry supervised layer, its outputs could sufficiently explain IT data.

Here in this commentary, we would say that this goal-driven approach, although with some notable successes and great potential for understanding sensory cortex, could be not as general as the authors (Yamins and DiCarlo, 2016) advocate, and its general use should be taken with special care. This is because as shown in Li et al. (2016), the 4 different HCNNs, with the same AlexNet architecture trained with the same dataset (ImageNet) but only from different random initializations, learned both convergent and divergent features although the 4 HCNNs have achieved the similar categorization performances: their top-1 accuracies are 58.65, 58.73, 58.79, and 58.84% respectively, which are also similar to the top-1 performance of 59.3% reported in the original study (Krizhevsky et al., 2012). In other words, some convergent features, which are individually similar or related via a linear transformation, are reliably learnt by the 4 HCNNs, yet other divergent features are not consistently learnt. In particular, the features at downstream layers are more divergent than convergent among the 4 HCNNs. The divergence is particularly marked by two aspects: (1) The responses of neurons at higher layers in one network were impossible to be linearly mapped to the responses of the neurons at the same layer in other networks (Table 1 in Li et al., 2016). Or the outputs of the neurons at the same layer in a pair of networks cannot be adequately related via a linear transformation; (2) Across different networks, their most active and least active filters (shown in Figures S11 and S12 in Li et al., 2016) were quite different, indicating different neuron selectivity. In sum, the results in Li et al. (2016) indicate that by merely optimizing the image categorization performances, different HCNNs can obtain different object representations but with similar categorization performance. This seems not consistent with the goal-driven principle.

In Hong et al. (2016), the authors were aware of this divergent HCNN learning problem. They said, quote: “It is not the case that any deep convolutional network trained to solve an arbitrary object categorization task will trivially exhibit the features of ventral stream that are produced in our original high-variation-trained computational model.” The authors seem to contribute such divergent-learning problems to the insufficiency of stimulus variations to stimulate IT neural sites. However, initialization is an inherent problem for HCNN learning, and it is not related to any external stimulus variations. In sum, HCNN architecture, initialization, learning algorithm, and training images all affect the outputs of the trained HCNN. We thought if different architectures are allowed, more divergent than convergent representations would be learnt, considering the existence of many local minima and the over-parameterization nature of HCNNs (LeCun et al., 2015). Hence “purely goal-driven” should be taken with great care in modeling sensory cortex.

## Author contributions

All authors listed have made a substantial, direct and intellectual contribution to the work, and approved it for publication.

### Conflict of interest statement

The authors declare that the research was conducted in the absence of any commercial or financial relationships that could be construed as a potential conflict of interest.
